# Higher Neutrophil-to-Albumin Ratio Is Associated with Greater Coronary Atherosclerotic Burden According to the Gensini Score in an Angiography-Referred Population

**DOI:** 10.3390/diagnostics16060864

**Published:** 2026-03-13

**Authors:** Ömer Faruk Çiçek, Ali Palice

**Affiliations:** 1 Department of Cardiology, Mehmet Akif Inan Training and Research Hospital, Sanliurfa 63040, Turkey; 2 Akçakale State Hospital, Sanliurfa 63500, Turkey; paliceali@gmail.com

**Keywords:** coronary artery disease, neutrophil-to-albumin ratio, Gensini score, inflammation

## Abstract

**Objective:** This study aimed to investigate the association between the neutrophil-to-albumin ratio (NAR) and coronary atherosclerotic burden, assessed using the Gensini score, in patients referred for coronary angiography (CAG). **Methods:** A total of 987 patients who underwent CAG at Sanliurfa Mehmet Akif Inan Training and Research Hospital between January 2020 and June 2023 were retrospectively analyzed. Demographic, clinical, and laboratory data, including complete blood count, albumin, lipid profile, and creatinine, were collected prior to angiography. The NAR was calculated as the ratio of absolute neutrophil count to serum albumin. Coronary atherosclerotic burden was assessed using the Gensini scoring system and analyzed across predefined score categories representing increasing anatomical disease extent: normal (score 0), mild (1–24), and severe (≥25). Statistical analyses included group comparisons and multivariable regression analyses appropriate to the study design. **Results:** Higher NAR values were associated with increased angiographic coronary atherosclerosis severity at the group level across Gensini score categories. This association remained statistically significant after prespecified multivariable adjustment for established cardiovascular risk factors. **Conclusions:** Higher NAR values were associated with greater angiographic coronary atherosclerotic burden, as quantified by the Gensini score, in an angiography-referred population.

## 1. Introduction

Coronary artery disease (CAD) is a leading cause of cardiovascular morbidity and mortality worldwide and represents a progressive inflammatory condition characterized by atherosclerotic involvement of the epicardial coronary arteries [1,2,3]. Although CAD may remain clinically silent for prolonged periods, acute plaque destabilization can precipitate ischemic events such as unstable angina or myocardial infarction [4,5]. Given the heterogeneity in disease progression and outcomes, accurate characterization of angiographic atherosclerotic burden in patients undergoing coronary angiography (CAG) is essential for optimizing preventive and therapeutic strategies [6].

Atherosclerosis is now widely recognized as an inflammation-driven process operating throughout the disease continuum, from early lesion development to plaque rupture [2]. Circulating immune cells, including neutrophils, lymphocytes, and monocytes, contribute to endothelial injury, plaque progression, and vascular instability [1,3]. Accordingly, complete blood count-derived inflammatory indices have gained interest as inexpensive and readily accessible markers reflecting systemic inflammatory activity in CAD [7,8].

Serum albumin, beyond its nutritional significance, exerts antioxidant and anti-inflammatory effects and has been associated with adverse cardiovascular outcomes when reduced [9]. Coronary atherosclerosis therefore reflects the dynamic balance between inflammatory injury mediated by circulating immune cells and vascular protective mechanisms linked to albumin. Indices capturing this balance may offer a more integrated representation of angiographic coronary atherosclerotic burden than evaluation of either component alone.

The neutrophil-to-albumin ratio (NAR), which combines an indicator of inflammatory activation (neutrophil count) with a marker of inflammatory–nutritional status (albumin), has recently emerged as a novel biomarker in various clinical settings [9,10,11]. Unlike traditional inflammatory markers that primarily reflect isolated inflammatory pathways or acute-phase responses, the NAR simultaneously reflects immune cell-mediated inflammatory activity and albumin-associated antioxidant and anti-inflammatory effects.

Although several studies have investigated inflammation-based indices in relation to CAD severity, evidence specifically examining the association between the NAR and angiographically assessed coronary atherosclerotic burden using validated scoring systems remains limited [7,10,12]. Most prior investigations have focused on prognostic endpoints or acute coronary syndromes rather than anatomically quantified coronary atherosclerosis. Given the routine availability and low cost of neutrophil and albumin measurements in daily clinical practice, further evaluation of the NAR as a laboratory correlate of anatomically defined coronary disease severity in angiography-referred populations appears warranted.

Accordingly, this study aimed to examine the association between the NAR and coronary atherosclerotic burden, as assessed by the Gensini score, in an angiography-referred population.

## 2. Materials and Methods

### 2.1. Study Population

This retrospective, single-center study included consecutive patients who underwent CAG at the Cardiology Clinic of Sanliurfa Mehmet Akif Inan Training and Research Hospital between January 2020 and June 2023. The study population consisted of adult patients (≥18 years) who underwent invasive CAG for suspected CAD. Patients with acute coronary syndrome were excluded from the study to minimize the confounding effects of acute systemic inflammation and to focus on a more homogeneous population with stable CAD. Patients with previously documented CAD, chronic inflammatory or autoimmune diseases, active infections, hematologic malignancies, severe valvular heart disease, hypertrophic cardiomyopathy, or incomplete medical records were also excluded. Accordingly, the study cohort was intentionally derived from patients undergoing CAG, representing a population with a high pre-test probability of CAD.

### 2.2. Ethical Approval

This study was conducted in accordance with the principles of the Declaration of Helsinki and approved by the Harran University Clinical Research Ethics Committee (approval date: 4 September 2023; approval number: HRÜ/23.16.01). Due to the retrospective design of the study, the requirement for informed consent was waived by the ethics committee.

### 2.3. Coronary Angiography and Gensini Score

CAG was performed by experienced interventional cardiologists using standard Judkins or radial access techniques. Angiographic images were independently evaluated by two experienced cardiologists who were blinded to the clinical and laboratory data according to predefined Gensini scoring criteria. In cases of discrepancy, assessments were jointly re-evaluated and resolved by consensus.

The angiographic severity and extent of coronary atherosclerosis were quantified using the Gensini scoring system, which integrates both the degree of luminal stenosis and the anatomical importance of the affected coronary segments. For each lesion, a severity score was assigned according to the percentage of diameter narrowing and multiplied by a segment-specific weighting factor reflecting the myocardial territory supplied, with higher total scores indicating a greater angiographic coronary atherosclerotic burden. In this study, coronary atherosclerotic burden refers to the anatomical extent and severity of coronary artery involvement as quantified using the Gensini score.

Based on the distribution of Gensini scores in the study population, patients were stratified into three predefined categories reflecting increasing angiographic atherosclerotic burden: Gensini score = 0, low burden (score 1–24), and high burden (score ≥25). This stratification was informed by the median Gensini score within the study population and was applied to facilitate analytical comparisons across increasing levels of angiographic burden.

### 2.4. Laboratory Assessments

Venous blood samples were collected from all participants prior to CAG following overnight fasting. All laboratory measurements were obtained from routine blood samples collected after hospital admission and prior to CAG, before any procedural intervention. Complete blood count, serum albumin, lipid profile, and creatinine levels were measured using standardized automated analyzers in the hospital’s central laboratory as part of routine clinical evaluation. Estimated glomerular filtration rate (eGFR, mL/min/1.73 m^2^) was calculated using the CKD-EPI equation based on routinely available serum creatinine, age, and sex data. The NAR was calculated by dividing the absolute neutrophil count (×10^9^/L) by the serum albumin concentration expressed in g/dL. Albumin values were originally reported in g/L by the institutional laboratory system and were converted to g/dL (g/L ÷ 10) prior to NAR calculation. The neutrophil-to-lymphocyte ratio (NLR) was calculated as the absolute neutrophil count divided by the absolute lymphocyte count. The systemic immune-inflammation index (SII) was calculated as platelet count × neutrophil count/lymphocyte count. The systemic inflammation response index (SIRI) was calculated as neutrophil count × monocyte count/lymphocyte count. All cell counts were obtained from the same complete blood count analysis.

### 2.5. Statistical Analysis

Data analyses were performed using the Statistical Package for the Social Sciences (SPSS), version 24. Continuous variables are summarized as the mean ± standard deviation, and categorical variables are expressed as frequencies and percentages. Group comparisons were conducted using appropriate parametric statistical tests according to the number of comparison groups, with post hoc analyses applied when necessary to account for unequal group sizes using Hochberg’s GT2 test. The relationship between the NAR and the Gensini score was evaluated using Spearman correlation analysis.

The primary endpoint of the multivariable analysis was angiographic coronary atherosclerotic burden, modeled as an ordinal outcome based on increasing Gensini score categories. The variables included in the multivariable ordinal logistic regression model were selected a priori based on established clinical relevance and the previous literature rather than solely on univariate statistical significance. To minimize the risk of model overfitting, the number of covariates was limited relative to the sample size, and no automated stepwise variable selection procedures were applied. Multicollinearity among covariates was assessed using variance inflation factors (VIFs), with values below 5 considered acceptable.

Multivariable ordinal logistic regression analysis was conducted to identify independent predictors of angiographic CAD severity. The proportional odds assumption was assessed and found to be satisfied for the ordinal logistic regression model. In this model, odds ratios (ORs) for the NAR were calculated per 0.1-unit increase to provide a clinically meaningful interpretation within the observed range (0.9–1.4). A *p*-value < 0.05 was considered statistically significant for all analyses. A post hoc power analysis based on the observed correlation between the NAR and the Gensini score (r = 0.46, *n* = 987) demonstrated a statistical power greater than 0.99 at a two-sided α level of 0.05, indicating adequate statistical power for the primary analyses.

## 3. Results

**Figure 1:**
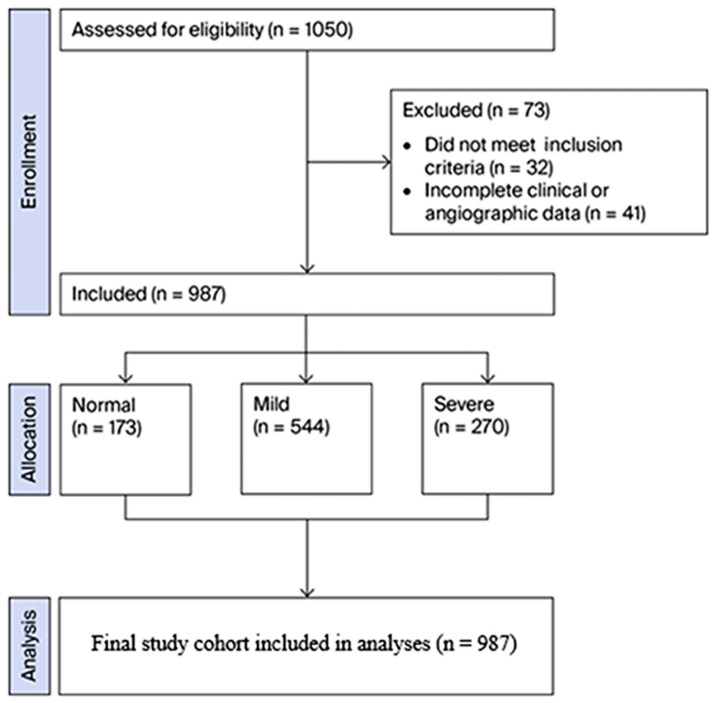
Flowchart illustrating patient selection, exclusion criteria, and final study population stratified according to the Gensini score categories.

Among the records reviewed, 1050 patients were initially assessed for eligibility. Following the application of the predefined exclusion criteria and the removal of cases with incomplete clinical or angiographic information, the final study population comprised 987 patients (Figure 1). Demographic characteristics across groups stratified by increasing Gensini score categories showed variation in sex distribution and the prevalence of diabetes mellitus (DM) and hypertension (HT). Laboratory parameters, including albumin, neutrophil count, monocyte count, platelet count, low-density lipoprotein cholesterol, creatinine, and the NAR, also varied across angiographic severity categories (Table 1). In contrast, lymphocyte counts appeared comparable across the Gensini score groups.

**Figure 2:**
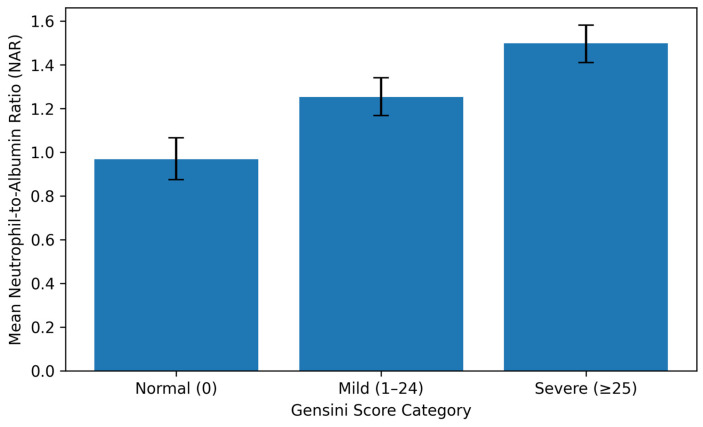
Distribution of neutrophil-to-albumin ratio values across angiographic coronary atherosclerosis severity categories defined by the Gensini score.

The NAR increased across angiographic Gensini score categories. The mean NAR was 0.97 (95% CI, 0.87–1.07) in the normal group, 1.25 (95% CI, 1.17–1.34) in the mild group, and 1.51 (95% CI, 1.42–1.59) in the severe group (Figure 2). Post hoc pairwise comparisons further demonstrated significant differences in NAR values between the Gensini score categories (Table 2, *p* < 0.001).

The figure illustrates group-level distributions of observed NAR values without implying diagnostic, predictive, or classification performance.

**Table 2:** NAR values across the Gensini score categories and post hoc pairwise comparisons.

					Hochberg’s GT2	
Gensini Score Group	*N*	Mean	SD	*p*	Gensini Score Group	*p*-Value
Normal	173	0.97	0.30	<0.001	Mild	<0.001
					Severe	
Mild	544	1.25	0.39		Normal	<0.001
					Severe	
Severe	270	1.51	0.47		Normal	<0.001
					Mild	

SD, standard deviation.

In the multivariable ordinal logistic regression model, the NAR was significantly associated with angiographic coronary atherosclerotic burden, as reflected by increasing Gensini score categories, after adjustment for traditional cardiovascular risk factors and renal function assessed by estimated glomerular filtration rate (eGFR) (per 0.1-unit increase; β = 0.058, SE = 0.011; OR 1.06, 95% CI 1.04–1.08, *p* < 0.001). DM (β = 0.51, SE = 0.22; OR 1.67, 95% CI 1.08–2.57, *p* = 0.019) and HT (β = 0.47, SE = 0.20; OR 1.60, 95% CI 1.09–2.35, *p* = 0.014) were also significantly associated with higher angiographic coronary atherosclerotic burden categories. Lower estimated glomerular filtration rate was modestly associated with greater CAD burden (per 10 mL/min/1.73 m^2^ decrease; β = 0.12, SE = 0.05; OR 1.13, 95% CI 1.02–1.26, *p* = 0.021). Male sex showed a borderline association with angiographic CAD severity (OR 1.43, 95% CI 0.98–2.10, *p* = 0.056), whereas LDL cholesterol (OR 1.04, 95% CI 0.99–1.10, *p* = 0.12) and age were not significantly associated with angiographic disease burden (Table 3).

Additional complete blood count-derived inflammatory indices, including the NLR, the SII, and the SIRI, were summarized across Gensini score categories (Table 4). Mean values of the NAR, the NLR, the SII, and the SIRI increased progressively from the normal to the severe angiographic burden category.

The NAR was independently associated with increasing angiographic coronary atherosclerotic burden in all comparative models (Table 5). This association remained significant after the inclusion of other inflammatory indices, including the NLR, the SII, and the SIRI. In contrast, these indices were not independently associated with angiographic disease burden when evaluated alongside the NAR. All analyses were adjusted for age, sex, DM, and HT.

**Table 4:** Distribution of inflammatory indices across angiographic coronary atherosclerotic burden categories defined by the Gensini score.

Variable	Normal (0)	Mild (1–24)	Severe (≥25)
NAR (mean ± SD)	0.97 ± 0.30	1.25 ± 0.39	1.51 ± 0.47
NLR (mean ± SD)	1.97 ± 0.76	2.65 ± 1.66	3.21 ± 1.58
SIRI (mean ± SD)	1.21 ± 0.63	1.77 ± 1.19	2.37 ± 1.40
SII (mean ± SD)	531.17 ± 256.43	629.85 ± 378.16	722.10 ± 456.97

Abbreviations: NAR, neutrophil-to-albumin ratio; NLR, neutrophil-to-lymphocyte ratio; SII, systemic immune-inflammation index; SIRI, systemic inflammation response index.

**Table 5:** Comparative multivariable analysis of the neutrophil-to-albumin ratio (NAR) and other inflammatory indices.

Model	NAR (per 0.1-Unit Increase) OR (95% CI)	*p*-Value	Other Inflammatory Index OR (95% CI)	*p*-Value
NAR only	1.22 (1.13–1.30)	<0.001	—	—
NAR + NLR	1.20 (1.11–1.29)	<0.001	1.11 (0.91–1.35)	0.319
NAR + SII	1.26 (1.16–1.36)	<0.001	0.74 (0.52–1.06)	0.100
NAR + SIRI	1.20 (1.10–1.31)	<0.001	1.10 (0.75–1.61)	0.637

NAR, neutrophil-to-albumin ratio; NLR, neutrophil-to-lymphocyte ratio; SII, systemic immune-inflammation index; SIRI, systemic inflammation response index; OR, odds ratio; CI, confidence interval.

## 4. Discussion

This study demonstrated that elevated NAR levels were significantly associated with angiographic coronary atherosclerotic burden assessed using the Gensini score. This association remained statistically independent after adjustment for conventional cardiovascular risk factors. Given the retrospective design, the findings should be interpreted as associative rather than causal. Although inter-individual overlap limits its discriminatory value at the individual level, the consistent stepwise increase across Gensini categories supports its interpretation as a population-level correlate of anatomical disease severity.

CAG remains the reference standard for assessing the anatomical extent of coronary atherosclerosis and guiding revascularization strategies. Among available scoring systems, the Gensini score provides a weighted quantification of stenosis severity and lesion location and has been widely used in biomarker-based investigations [13]. Prior studies have demonstrated associations between Gensini-defined disease severity and laboratory-derived inflammatory markers in both acute and stable CAD populations [14,15]. Our results extend this evidence by positioning the NAR specifically within an angiography-referred cohort evaluated primarily at the anatomical level rather than through outcome-driven endpoints. Recent studies have further demonstrated associations between the NAR and other forms of atherosclerotic burden, including carotid atherosclerosis, supporting its broader relevance as a vascular inflammatory marker [16].

Atherosclerosis is a chronic inflammatory process characterized by endothelial dysfunction and immune activation [1,2,3]. Circulating inflammatory cells, particularly neutrophils, contribute to vascular injury through oxidative and proteolytic mechanisms [2,17]. Serum albumin, in contrast, exerts antioxidant and anti-inflammatory effects and has been consistently associated with adverse cardiovascular outcomes when reduced [9]. Within this biological framework, the NAR integrates inflammatory activation and diminished vascular protective capacity into a single composite index [7,12]. This combined inflammatory–nutritional profile may explain the persistent independent association between NAR and angiographic coronary atherosclerotic burden observed in the multivariable analysis.

In addition to the NAR, other complete blood count-derived inflammatory indices—including NLR, SII, and SIRI—showed similar stepwise increases across Gensini score categories, consistent with inflammation-related patterns previously described in cardiovascular disease [15,18,19]. The NAR may therefore be considered a pragmatic and readily available indicator of systemic inflammatory activity and endothelial dysfunction. While several inflammatory indices increased with angiographic severity in unadjusted analyses, only the NAR remained independently associated with coronary atherosclerotic burden when evaluated alongside NLR, SII, and SIRI. This comparative observation suggests that, among commonly used inflammation-derived markers, NAR may capture a more stable inflammatory–nutritional signal linked specifically to anatomically defined coronary atherosclerosis, constituting the principal contribution of the present study.

Most previous studies evaluating inflammatory or nutritional biomarkers in CAD have focused on outcome-driven or disease-specific populations, where biomarker levels may be influenced by acute events or therapeutic interventions [17,20]. In contrast, the angiography-referred population examined in the present study allows inflammatory–nutritional balance to be evaluated directly at the level of coronary anatomy, independent of downstream clinical outcomes. By aligning inflammatory status directly with anatomical burden, this design strengthens interpretation of NAR as an anatomical correlate rather than a prognostic or event-driven marker.

In the multivariable analysis, traditional risk factors such as age, sex, and LDL cholesterol did not show independent associations with angiographic coronary atherosclerotic burden. This finding likely reflects the relatively homogeneous and risk-enriched characteristics of an angiography-referred population. Adjustment for overlapping cardiometabolic variables may have reduced the detectable independent effect of age and other classical risk factors. These results indicate that the association between NAR and angiographic burden was independent of conventional risk factors within this cohort.

This study therefore provides angiography-based evidence supporting an association between the NAR and objectively quantified coronary atherosclerotic burden in an angiography-referred population, further delineating its relevance as an integrated inflammatory–nutritional marker that reflects anatomical disease severity at the population level.

The study has several limitations. Its retrospective, single-center design may limit external validity and introduce selection bias. Despite multivariable adjustment, residual confounding cannot be fully excluded due to unavailable variables such as body mass index, statin or anti-inflammatory therapy. Temporal changes in NAR were not evaluated, and causal inference is not possible. The Gensini score reflects anatomical luminal narrowing without functional ischemic assessment, and formal inter-observer reliability metrics were not calculated; therefore, findings should be interpreted at the group level. As the cohort consisted predominantly of patients with mild angiographic disease undergoing CAG, generalizability to unselected populations may be limited.

## 5. Conclusions

In conclusion, higher NAR values were consistently associated with greater angiographic coronary atherosclerotic burden, as quantified by the Gensini score, in an angiography-referred population. The observed association remained robust after adjustment for established cardiovascular risk factors and renal function. However, the present findings do not support the use of NAR as a diagnostic or clinical decision-making tool at the individual patient level. In this context, while other inflammation-derived indices lost their independent associations, the demonstration of a graded and independent relationship between NAR and angiographic disease burden highlights NAR as a stable laboratory correlate of coronary anatomical severity at the population level. These findings provide an angiography-based reference framework that may inform future prospective studies aimed at standardizing NAR categorization and evaluating its relevance in relation to functional ischemia or long-term clinical outcomes.

## Figures and Tables

**Table 1:** Demographic, clinical, and laboratory characteristics of the study population.

Variable	Overall Population (*n* = 987)	Normal (*n* = 173)	Mild CAD (*n* = 544)	Severe CAD (*n* = 270)
Age (years), mean ± SD	58.20 ± 10.21	55.52 ± 8.62	60.45 ± 8.24	58.78 ± 10.61
Sex (male/female)	523/464	69/104	290/254	164/106
Diabetes mellitus, *n* (%)	147 (14.9%)	5 (2.9%)	108 (19.9%)	34 (12.6%)
Hypertension, *n* (%)	318 (32.2%)	23 (13.3%)	188 (34.6%)	107 (39.6%)
Smoking, *n* (%)	230 (23.3%)	41 (23.7%)	107 (19.7%)	82 (30.4%)
Albumin, g/L	40.70 ± 3.50	44.39 ± 2.43	40.96 ± 3.57	39.5 ± 2.91
Neutrophil count, ×10^9^/L	5.34 ± 1.69	4.28 ± 1.26	5.25 ± 1.53	5.81 ± 1.7
Lymphocyte count, ×10^9^/L	2.21 ± 0.87	2.3 ± 0.63	2.26 ± 0.87	2.21 ± 0.97
Monocyte count, ×10^9^/L	0.69 ± 0.23	0.6 ± 0.19	0.67 ± 0.24	0.72 ± 0.24
Platelet count, ×10^9^/L	239.21 ± 70.71	272.86 ± 71.22	241.99 ± 64.47	234.91 ± 80.34
LDL-C mg/dL	117.20 ± 35.83	106.86 ± 32.35	118.07 ± 35.98	119.58 ± 34.91
Creatinine, mg/dL	0.91 ± 0.21	0.84 ± 0.22	0.91 ± 0.22	0.92 ± 0.20
NAR (neutrophil ×10^9^/L/albumin g/dL)	1.33 ± 0.46	0.97 ± 0.30	1.25 ± 0.39	1.51 ± 0.47
Gensini score	29.07 ± 25.49	0	14.84 ± 7.41	45.28 ± 21.49

Numerical variables are shown as mean ± standard deviation. Categorical variables are presented as number (percentage). Abbreviations: SD, standard deviation; LDL-C, low-density lipoprotein cholesterol; NAR, neutrophil-to-albumin ratio; CAD: coronary artery disease. Albumin is presented in g/L, as reported by the laboratory; NAR values are calculated using albumin converted to g/dL.

**Table 3:** Multivariable ordinal logistic regression analysis of the association between the neutrophil-to-albumin ratio (NAR) and angiographic CAD burden based on the Gensini score.

Variable	β (SE)	OR (95% CI)	*p*-Value
NAR (per 0.1-unit increase)	0.058 (0.011)	1.06 (1.04–1.08)	<0.001
LDL-C (mg/dL, per 10 mg/dL)	0.04 (0.03)	1.04 (0.99–1.10)	0.100
Age (years)	0.02 (0.01)	1.02 (0.99–1.04)	0.110
Male sex	0.36 (0.19)	1.43 (0.98–2.10)	0.056
Diabetes mellitus	0.51 (0.22)	1.67 (1.08–2.57)	0.020
Hypertension	0.47 (0.20)	1.60 (1.09–2.35)	0.015
eGFR (per 10 mL/min/1.73 m^2^ decrease)	0.12 (0.05)	1.13 (1.02–1.26)	0.021

Dependent variable: Gensini score category reflecting angiographic CAD burden (normal/mild/severe). Abbreviations: NAR, neutrophil-to-albumin ratio; LDL-C, low-density lipoprotein cholesterol; OR, odds ratio; CI, confidence interval; SE, standard error; eGFR, estimated glomerular filtration rate.

## Data Availability

The data presented in this study are available from the corresponding due to patient privacy and ethical considerations.
